# REM‐OSA vs NREM‐OSA in Children: Separate Clinical Phenotypes or Markers of OSA Severity

**DOI:** 10.1002/ppul.71399

**Published:** 2025-11-23

**Authors:** Haneen Toma, Amal Alnaimi, Mutasim Abu‐Hasan, Antonisamy Belavendra, Ibrahim Janahi

**Affiliations:** ^1^ Department of Pediatric Medicine Division of Pulmonary Doha Qatar; ^2^ Weill Cornel Medicine‐Qatar (WCM‐Q), Qatar Foundation Doha Qatar

**Keywords:** Non‐ Rapid Eye Movement (NREM), NREM‐OSA, obstructive sleep apnea (OSA), polysomnography (PSG), Rapid Eye Movement (REM), REM‐OSA

## Abstract

**Introduction:**

Obstructive sleep apnea (OSA) occurs predominantly during REM sleep (REM‐OSA) compared to NREM sleep (NREM‐OSA) in both children and adults. However, it is not clear whether REM‐OSA and NREM‐OSA are separate clinical phenotypes of OSA or represent 2 ends of OSA severity spectrum.

**Objective:**

We aimed to compare clinical and polysomnography (PSG) characteristics of REM‐OSA versus NREM‐OSA in children, and to evaluate the effect of these characteristics on REM‐related over NREM‐related obstructive AHI ratio (REM‐OAHI/NREM‐OAHI).

**Methods:**

Clinical and PSG data of all children diagnosed with moderate to severe OSA by PSG between 2019 and 2024 were collected and analyzed. REM‐OSA was defined as REM‐OAHI/NREM‐OAHI ratio of ≥2. NREM‐OSA was defined as REM‐OAHI/NREM‐OAHI of < 2.

**Results:**

A total of 253 patients (169 male and 84 female) met the inclusion criteria. REM‐OSA was identified in 174 (68.7%) patients, and NREM‐OSA in 79 (31.2%) patients. There was no significant difference between REM‐OSA and NREM OSA groups in gender, BMI, clinical diagnosis, symptoms or oxygenation indices. However, REM‐OSA group had lower OAHI and less severe OSA than NREM‐OSA group (*p* = 0.004, *p* = 0.003, respectively). Regression analysis showed that low OAHI was the only significant predictor of REM‐OSA and the only predictor of high REM‐AHI/NREM‐AHI ratio, respectively (*p* = 0.001, *p* = 0.001).

**Conclusion:**

REM‐OSA and NREM‐OSA are not likely to be separate clinical phenotypes of OSA but only markers of the disease severity, with NREM‐OSA indicating more severe disease.

**Abbreviations**
NREM‐OSA: Non‐ Rapid Eye Movement Obstructive Sleep Apnea
OSA: obstructive sleep apnea
PSG: Polysomnography
REM‐OSA: Rapid Eye Movement Obstructive Sleep Apnea

## 1 Introduction

Obstructive sleep apnea (OSA) is the most common sleep related breathing disorder in children. OSA is characterized by intermittent partial or complete obstruction of the upper airways during sleep which disrupts sleep and/or gas exchange. The prevalence of OSA in children ranges from 1.2% to 5.7% [1]. Polysomnography (PSG) is the gold standard diagnostic test for OSA. Diagnoses is conventionally based on the obstructive apnea and hypopnea index (OAHI) which is defined as the number of these events per hour of sleep time.

In children, OSA occurs more frequently during REM sleep. REM‐related obstructive events are longer in duration and more likely to be associated with low oxygen desaturation compared to NREM‐related obstructive events [2]. REM‐predominant OSA (REM‐OSA) is conventionally defined by REM‐OAHI/NREM‐OAHI ratio cut off value of ≥2, while NREM‐predominant OSA (NREM‐OSA) is defined by a cut off value of < 2 events/hour. Previous studies showed that the prevalence of REM‐OSA in children is very high, ranging from 69.6% to 74.7% [3, 4]. Several physiological factors contribute to the high prevalence of REM‐OSA including the low muscle tone, decreased ventilatory response to hypoxia and hypercapnia, and the low arousal threshold during REM [3].

REM‐OSA has been extensively studied in children not only because of its high prevalence but because of its clinical significance as well. REM‐OSA has disruptive effect on the sleep architecture and reduces REM due to frequent arousals. REM sleep plays critical role in memory formation, emotional processing, neuronal plasticity, and the overall brain function in children. REM‐OSA correlates with negative neurocognitive outcomes [5]. Also, REM‐OSA is associated with increased sympathetic activity and elevated cardiovascular risk including high nocturnal blood pressure [6, 7].

On the other hand, NREM‐OSA is far less prevalent than REM‐OSA in children. The clinical significance of NREM‐sleep is not well understood. It is debatable whether NREM‐OSA is a separate clinical phenotype of OSA, or if REM‐OSA and NREM‐OSA are only markers of OSA severity, and which end of the disease severity spectrum does REM‐OSA and NREM‐OSA represent. For the reasons explained above, it has been argued that REM‐OSA represents severe OSA. On the other hand, it can be argued that REM‐OSA reflects milder OSA since obstructive events tend to occur only during REM sleep in mild OSA because of the airway hypotonia and the blunted the respiratory responses. However, as the upper airway obstruction becomes more severe, the frequency of obstructive events during NREM sleep increases. Since NREM sleep is much longer than REM sleep, the increased frequency of NREM events in severe OSA leads to lower REM‐OAHI/NREM‐OAHI ratio. In this case, NREM‐OSA becomes the marker of more severe degree of OSA.

Several adult studies have shown that NREM‐OSA correlate with more severe disease as compared to REM‐OSA [8, 9]. However, fewer studies have evaluated disease severity and clinical determinants of REM‐OSA versus NREM‐OSA in children. Only one study suggested that REM‐OSA is associated with more severe OSA [1]. Rest of pediatric studies suggest that REM‐OSA and NREM‐OSA have no correlation with OSA severity, but represent two separate clinical phenotypes of OSA [7, 10, 11]. Pediatric studies differ greatly in their study population and methodology, and they generally include limited clinical and sleep variables. All pediatric studies used the conventional REM‐OAHI/NREM‐OAHI ratio of ≥2 as the accepted cut off value to discriminate between the two types of OSA. However, this widely used cut off value is completely arbitrary and has not been clinically validated. None of these studies used REM‐OAHI/NREM‐OAHI ratio as a continuous outcome variable in patients with OSA.

In this study we aim to retrospectively evaluate clinical and sleep‐related determinants of REM‐OSA versus NREM‐OSA. We also aimed to evaluate the effect of these clinical and PSG variables on REM‐OAHI/NREM‐OAHI ratio as a continuous variable. We hypothesized that REM‐OSA and NREM‐OSA are not separate clinical phenotypes of OSA but markers of OSA severity, with REM‐OSA indicating less severe disease than NREM‐OSA.

## 2 Methods

We retrospectively analyzed all available clinical and PSG data of children (< 18 years) who were diagnosed with OSA between September 1, 2019, and May 23, 2024 at Sidra Medicine Pediatric Sleep lab, Doha, Qatar. All PSG studies were performed using Sleepwear G3 software (Alice 6, Philips). Sleep stages and respiratory events were scored according to the American Academy of Sleep Medicine (AASM) scoring manuals (2023) [12, 13]. OSA diagnosis was confirmed if obstructive AHI (OAHI) was ≥1.5 events per hour. OSA was considered mild if OAHI was > 1.5 but < 5 events/our, moderate if OAHI was > 5 but < 10 events/hour, and severe if OAHI was > 10 events/hour [13]. Only children with sleep efficiency of > 40% and who have moderate to severe OSA were included in the analysis. The inclusion of patients with moderate to severe OSA only was made in attempt to eliminate data noise in patients with mild OSA which can confound possible significant correlations. REM‐OSA was defined as REM‐OAHI/NREM‐OAHI ratio ≥2. NREM‐OSA was defined as REM‐OAHI/NREM‐OAHI ratio < 2.

Demographic and clinical data were extracted from the hospital electronic medical records. We compared children with REM‐OSA versus NREM‐OSA groups in terms of age, gender, BMI, clinical diagnosis, sleep‐related symptoms, REM%, AHI, OAHI, average O2 saturation, nadir O2 saturation, and sleep time spent with O2 saturation below 90%. We evaluated the same parameters as predictors of REM‐OSA using logistic regression analysis. We then evaluated the same parameters as predictors of REM‐OAHI/NREM OAHI as a continuous variable using linear regression analysis.

To compare our findings with published studies, we conducted literature review of all pediatric studies evaluating clinical and PSG determinants of REM‐OSA versus NREM‐OSA in children. Patient population, methodology and results of these studies were summarized, discussed and compared to our study.

The study was approved by the Research Ethics Board at Sidra Medicine, Doha, Qatar (IRB No. 2202346).

### 2.1 Statistical Analysis

Continuous variables were presented as mean ± SD if normally distributed and as median (IQR) if not normally distributed. Prevalence of REM‐OSA and NREM/OSA was calculated as number of REM‐OSA patients and NREM‐OSA patients over total number of study patients. Comparison between REM‐OSA and NREM‐OSA groups was performed using Chi square analysis for categorical variables, Mann‐Whitney for non‐parametric continuous variables and student *t*‐test for parametric continuous variables.

Multivariable Logistic regression analysis was used to evaluate the clinical and sleep‐related predictors of REM‐OSA as a categorical outcome. Potential predictors including age, gender, sleep related symptoms, clinical diagnosis, REM%, and AHI were examined. Multivariable linear regression analysis was also performed to evaluate the same predictors for REM‐OAHI/NREM‐OAHI ratio as a continuous variable. BMI was removed from both regression models because of high collinearity with age. Logarithmic transformation was applied for positively skewed variables in the regression analysis. *p* value < 0.05 was considered statistically significant.

## 3 Results

A total of 253 patients (169 males and 84 females) met the study inclusion criteria. Median (IQR) age was 7.6 years (3.4 to 13.5). Median (IQR) BMI was 22 kg/m² (16.6 to 37.8). At total of 82 (32.4%) patients were diagnosed with obesity, 36 (14.2%) patients with neuromuscular diseases, 53 (20.9%) patients with Down syndrome, 15 (5.9%) patients with Prader‐Willi syndrome, 36 (14.2%) patients with other syndromes, and 31 (12%) patients with no comorbidities. Summary of demographics and clinical characteristics are presented in Table 1.

**Table 1:** Baseline demographic and clinical characteristics in all patients, REM‐OSA and NREM‐OSA patients.

	REM‐OSA	NREM‐OSA	Total	Test†
	(*n* = 174)	(*n* = 79)	(*n* = 253)	
Age (years)^a^	6.3 (2.8, 12.4)	9.5 (5.5, 14.2)	7.6 (3.4, 13.5)	0.004
Gender, *n* (%)				
Male	112 (64.4)	57 (72.2)	169 (66.8)	0.223
Female	62 (35.6)	22 (27.8)	84 (33.2)	
BMI(k/m^2^)^a^	22.0 (16.7, 37.8)	24.2 (16.6, 37.6)	22.0 (16.6, 37.8)	0.940
Symptoms, *n* (%)				
Snoring and witnessed apnea	74 (43.0)	34 (43.6)	108 (43.2)	0.841
Snoring only	50 (29.1)	25 (32.1)	75 (30.0)	
Witnessed apnea only	5 (2.9)	1 (1.3)	6 (2.4)	
Neither snoring nor witnessed apnea	43 (25.0)	18 (23.1)	61 (24.4)	
Diagnosis, *n* (%)				
Snoring	26 (14.9)	5 (6.3)	31 (12.3)	0.089
Obesity	55 (31.6)	27 (34.2)	82 (32.4)	
NMD	26 (14.9)	10 (12.7)	36 (14.2)	
DS	29 (16.7)	24 (30.4)	53 (20.9)	
PWS	12 (6.9)	3 (3.8)	15 (5.9)	
Others	26 (14.9)	10 (12.7)	36 (14.2)	

Abbreviations: BMI, body mass index; DS, down syndrome; NMD, neuro muscular disease; PS, prader willi syndrome.

^a^ Median(IQR).

^†^ Man–Whitney *U*‐test or chi‐square test.

A total of 90 (35.6%) patients had moderate OSA, while 163 (64.4%) had severe OSA. Median obstructive apnea‐hypopnea index (OAHI) was 11 events per hour (IQR: 7.2 to 18.7). Median REM apnea‐hypopnea index (REM‐AHI) was 29 events/hour (IQR: 16.8 to 44). Median NREM‐ AHI was 6.7 events/hour (IQR: 3.6 to 13.3) (Table 2).

**Table 2:** Polysomnography data in all patients, REM‐OSA and NREM‐OSA patients.

Variables	REM‐OSA	NREM‐OSA	Total	Test†
	(*n* = 174)	(*n* = 79)	(*n* = 253)	
Sleep efficiency^a^	75.7 (63.6, 86.0)	79.0 (63.5, 84.6)	76.8 (63.6, 86.0)	0.910
Sleep onset^a^	20.5 (6.0, 49.1)	18.0 (7.0, 57.4)	19.9 (6.7, 50.5)	0.771
REM Latency^a^	98.5 (60.2, 161.0)	116.2 (73.5, 170.5)	101.8 (62.5, 163.5)	0.106
WASO^a^	80.8 (32.5, 132.5)	66.0 (43.0, 126.0)	72.0 (36.0, 127.0)	0.958
REM%^a^	22.0 (18.2, 27.8)	20.0 (15.7, 26.4)	21.1 (17.0, 27.4)	0.034
Snoring, *n* (%)				
Yes	125 (71.8)	56 (70.9)	181 (71.5)	0.876
No	49 (28.2)	23 (29.1)	72 (28.5)	
Severity, *n* (%)				
Moderate	69 (39.7)	21 (26.6)	90 (35.6)	0.044
Severe	105 (60.3)	58 (73.4)	163 (64.4)	
REM AHI^a^	33.0 (22.4, 50.4)	15.9 (9.3, 31.0)	29.0 (16.8, 44.0)	< 0.001
NREM AHI^a^	4.8 (2.7, 8.4)	14.3 (9.1, 29.0)	6.7 (3.6, 13.3)	< 0.001
OAHI^a^	9.9 (7.1, 16.5)	13.1 (8.3, 23.1)	11.0 (7.2, 18.7)	0.003
CAHI^a^	0.3 (0.0, 1.2)	0.2 (0.0, 0.8)	0.3 (0.0, 1.1)	0.227
Average O2^a^	96.0 (95.0, 97.0)	96.0 (94.0, 97.0)	96.0 (95.0, 97.0)	0.396
Nadir O2^a^	82.5 (76.0, 87.0)	86.0 (77.0, 88.0)	83.0 (76.0, 87.0)	0.044
% of total sleep time with SpO2 < 90%^a^	0.5 (0.1, 2.3)	0.4 (0.0, 5.3)	0.4 (0.0, 2.5)	0.951
ODI^a^	16.7 (10.6, 27.0)	20.0 (11.1, 39.0)	17.2 (10.8, 28.0)	0.157

^a^ Median(IQR)

^†^ Man–Whitney *U*‐test or chi‐square test. AHI, apnea hypopnea index; CAHI, central AHI; OAHI, Obstructive AHI; WASO, wakefulness after sleep onset.

REM‐OSA was present in 174 (68.7%) patients. NREM‐OSA was present in 79 (31.2%) patients. There was no significant difference between both groups in gender, BMI, clinical diagnosis, or sleep related symptoms. Age was lower in REM‐OSA group compared to NREM‐OSA group (*p* = 0.004). There was no significant difference between both groups in sleep efficiency.

NREM‐OSA group had lower REM% than REM‐OSA group (*p* = 0.034). NREM‐OSA group had significantly higher OAHI, higher REM‐OAHI and higher NREM‐OAHI than REM‐OSA (*p* = 0.003, *p* < 0.001, *p* < 0.001, respectively). Also, NREM‐OSA group had higher OSA severity than REM‐OSA group (*p* = 0.044). There was no significant difference between both groups in average O2% saturation or in percentage of total sleep time spent with O2 saturation below 90%. However, median O2% saturation nadir was lower in REM‐OSA group compared to NREM‐OSA (*p* = 0.044) (Table 2).

Logistic regression analysis showed low OAHI as the only significant predictor of REM‐OSA (OR = 0.51, *p* = 0.003) (Table 3). Multivariable linear regression analysis showed low OAHI as the only significant predictor of high REM‐AHI/NREM‐AHI ratio (OR = 0.61, *p* < 0.001) (Table 4).

**Table 3:** Logistic regression analysis of REM‐OSA as dependent variable.

Variables	Univariate				Multivariable			
	OR	95% CI		*p* value	OR	95% CI		*p* value
		Lower limit	Upper limit			Lower limit	Upper limit	
Age (years)	0.93	0.88	0.98	0.007	0.96	0.9	1.0	0.113
Gender (female)	1.43	0.8	2.6	0.224	1.42	0.8	2.6	0.259
Symptomatic (yes)	1.11	0.6	2.1	0.740	0.93	0.5	1.8	0.839
Diagnosis (yes)	0.39	0.1	1.0	0.060	0.56	0.2	1.5	0.257
REM	1.04	1.0	1.7	0.030	1.02	0.9	1.1	0.308
Obstructive apnea hypopnea index (OAHI)‐log transformed	0.49	0.32	0.73	< 0.001	0.51	0.33	0.77	0.003

**Table 4:** Linear regression analysis of REM‐AHI/NREM‐AHI ratio (log transformed) as a continuous.

Variables	Univariate				Multivariable			
	OR	95% CI		*p* value	OR	95% CI		*p* value
		Lower limit	Upper limit			Lower limit	Upper limit	
Age (years)	0.97	0.95	1.0	0.051	0.98	0.9	1.0	0.271
Gender (female)	1.19	0.9	1.6	0.282	1.19	0.8	1.6	0.270
Symptomatic (yes)	1.09	0.8	1.5	0.633	0.98	0.7	1.4	0.902
Diagnosis (yes)	0.82	0.5	1.3	0.372	0.98	0.6	1.5	0.923
REM	1.02	0.9	1.0	0.066	1.01	0.9	1.0	0.303
Obstructive apnea hypopnea index (OAHI)‐log transformed	0.59	0.48	0.74	< 0.001	0.61	0.49	0.76	< 0.001

## 4 Discussion

In this study, we evaluated the clinical and physiological determinants of REM‐OSA as compared to NREM‐OSA in children with moderate to severe OSA. We found that REM‐OSA is more prevalent in these children (68.7%) consistent with previous studies which showed that OSA is predominantly REM‐related in children, with prevalence rates ranging between 69.6% and 74.7% [4]. The high prevalence of REM‐OSA is attributed to decreased muscle tone, decreased ventilatory response to hypoxia and hypercapnia, and low arousal threshold during REM sleep compared to NREM sleep [3]. Furthermore, low lung volumes during REM sleep leads to pronounced oxygen desaturation during respiratory events [14]. Adult studies, on the other hand, have demonstrated lower prevalence of REM‐OSA than seen in pediatric population, ranging from 10% to 36% [15].

The main finding of the study was the higher OAHI, REM‐OAHI and NREM‐OAHI in NREM‐OSA group compared to REM‐OSA group. In addition, logistic regression analysis showed that low OAHI was the only predictor of REM‐OSA. Multivariate linear regression analysis showed that low OAHI was the only predictor of high REM‐OAHI/NREM‐OHI ratio. These findings strongly suggest that NREM‐OSA is associated with more severe OSA. These findings have significant clinical implications. The high association between NREM‐OSA and disease severity argues for the inclusion of REM‐OAHI/REM‐OAHI ratio as an added PSG parameter that provide further evidence to disease severity in addition to conventional parameters such as AHI, OAHI, and REM‐OAHI which can better inform treatment decisions. Future studies are needed to evaluate the effect of NREM‐OSA on long term clinical outcome.

On the other hand, there was also no difference between REM‐OSA and NREM‐OSA group in terms of sleep related symptoms or clinical diagnosis. None of the clinical characteristics of patients was predictive of REM‐OSA frequency or REM‐OAHI/NREM‐OAHI ratio. These findings suggest that REM‐OSA and NREM‐OSA do not represent two separate phenotypes of OSA but more likely reflect two ends of the spectrum of OSA disease severity.

Mild OSA occur primarily during REM because of the relatively lower upper airway muscle tone and the diminished ventilatory responses, but as the degree of airway obstruction increases, OSA becomes more frequent during both REM and NREM. The increase in NREM events explains the inverse association between OAHI and the REM‐OAHI/NREM‐OAHI ratio.

We found no significant difference between REM‐OSA and NREM OSA groups on average O2 saturation and % of sleep time spent with O2 saturation < 90% of sleep time. There was a strong association between OAHI and all oxygenating indices (Average O2 saturation, % of sleep times spent with O2 saturation < 90% and nadir O2 saturation). However, there was no significant association between OAHI and REM‐OAHI/NREM‐OAHI ratio (Table 5). These findings suggest that oxygenation indices are not affected directly by REM‐OSA and NREM‐OSA, but through OSA severity. Therefore, oxygenating indices were removed from the regression analysis of REM‐OSA and REM‐OAHI/NREM‐OAHI ratio due to the strong collinearity between these indices and OAHI.

**Table 5:** Spearman rank correlation between age, BMI, oxygen parameters, and OAHI.

	Age	BMI	AverageO2	NadirO2	% of total sleep time with SpO2 < 90%	OAHI
BMI	0.674 (*p* < 0.001)					
AverageO2	−0.105 (*p* = 0.101)	−0.136 (*p* = 0.032)				
NadirO2	0.149 (*p* = 0.019)	0.025 (*p* = 0.694)	0.489 (*p* < 0.001)			
% of total sleep time with SpO2 < 90%	−0.076 (*p* = 0.233)	0.024 (*p* = 0.708)	−0.692 (*p* < 0.001)	−0.809 (*p* < 0.001)		
OAHI	0.085 (*p* = 0.185)	0.102 (*p* = 0.109)	−0.298 (*p* < 0.001)	−0.407 (*p* < 0.001)	0.514 (*p* < 0.001)	
REM‐OAHI/NREM OAHI ratio	−0.156 (*p* = 0.014)	0.014 (*p* = 0.826)	0.086 (*p* = 0.179)	−0.092 (*p* = 0.148)	−0.092 (*p* = 0.148)	−0.278 (*p* < 0.001)

*Note:* Spearman rank, rho (*p* value).

Our findings of significant association between NREM‐OSA and severe OSA does not agree with previously published pediatric studies, most of which showed no difference in severity between REM‐OSA and NREM‐OSA (Table 6). One study by Wu et al. showed significant association between REM‐OSA and severe OSA, which is contrary to our study finding [10]. However, the study included younger patients with an average OAHI that is much lower than our study population, therefore, with milder OSA (Table 5). Furthermore, the study did not include patients with comorbidities as is the case with our study which can also explain the higher degree of OSA severity in our study.

**Table 6:** Summary of published pediatric studies evaluating determinants of REM‐OSA.

First author/year	Patient population	Number of the patients	Age,	Average OAHI	Definition of REM‐OSA	Results
Yunxiao Wu [1] /2022	Children 2–12 yrs. old with OSA.	474	4.8 (4.1, 6.4) median (IQR)	3.9 (1.9, 7.9) median (IQR)	REM‐OAHI/NREM‐OAHI ≥ 2	REM‐OSA is associated with younger age, higher AHI, low oxygen saturation and high OHI.
Surisa chammanpet [10] /2022	Children less than 18 years old with OSA.	366	8 (5, 12) median (IQR)	8.4 (4.9, 15.2) median (IQR)	REM‐OAHI/NREM‐OAHI ≥ 2	REM‐OSA is associated with female gender, low O2 saturation. REM OSA was not related to OSA severity.
Kate C Chan [7] /2021	Children 6–13 yrs. old with OSA.	610	REM‐OSA group: 10.2 (1.8) NREM‐OSA group: 9.9(1.8) Mean (SD)	REM‐OSA group: 2.47 (1.42, 4.50) NREM‐OSA group: 2.53 (1.54, 6.37) median (IQR)	REM‐OAHI/NREM‐OAHI ≥ 2,	REM‐OSA was associated with lower ODI. REM OSA was not related to OSA severity.
Nicole vergenis [11] /2009	Children less than 18 yrs. old with moderate to severe OSA.	142	5.6 (3.6) Mean (SD)		(Log REM ‐OHI + 0.5)/(Log NREM OHI + 0.5) = 0.51	REM OSA was associated with younger age, low O2 saturation, but not related to OSA severity.

Such differences in methodology can contribute to the seemingly contradictory conclusions between previous studies and our study. Similar to the study by Wu et al., majority of previous pediatric studies included children with mild OSA disease which can influence the correlation strength between REM‐OSA and OAHI. The inclusion of patients with less severe disease can introduce more data noise in the REM‐OAHI/NREM‐OAHI ratio. We elected to exclude patients with mild disease in our study to avoid data noise that can be introduced by these subjects, and to ensure statistical significance that reflects clinical significance. Moreover, previous pediatric studies also used REM‐OSA as a categorical outcome variable based on the cutoff value of 2 for REM‐OAHI/NREM‐OAHI ratio, which is an arbitrary value which has not been clinically validated. None of these studies investigated the ratio as a continuous variable. Unlike pediatric studies, majority of the adult studies showed conclusively that NREM‐OSA is associated with more severe OSA as demonstrated in our study [8, 9].

We found that NREM‐OSA group had shorter REM stages than REM‐OSA group. The short REM sleep can be due to sleep fragmentation caused by the frequent apnea events, a finding shown by adult studies which strongly suggest that severe OSA decreases the duration of REM sleep [16]. However, regression models in our study showed that the correlation between REM% and REM‐OSA, and the correlation between REM% an REM‐OAHI/REM‐OAHI ratio were not significant.

Even though minimum (nadir) oxygen saturation was significantly lower in the REM‐OSA group compared to the NREM‐OSA group, nadir O2 saturation does not necessarily reflect more OSA severe disease in children. Nadir O2 is highly affected by motion artifacts especially in children. Moreover, nadir O2 saturation was not a significant predictor in the regression analysis models. On the other hand, average O2 saturation and percentage of TST spent below 90%, which are more accurate measures of oxygenation, were not different between both groups. All oxygen indices did not significantly correlate with REM‐OSA or with REM‐OAHI/NREM‐OAHI ratio after adjusting for OAHI, due to the strong collinearity between O2 saturation indices and OAHI as explained above. Therefore, they were removed from the regression models.

Previous pediatric studies have reported lower O2 saturation indices in REM‐OSA than in NREM‐OSA (Table 6). However, none of these studied have adjusted for OSA severity when comparing between both groups. Moreover, none of these studies evaluated the association between oxygenations indices and REM‐AHI/NREM AHI as continuous variable as done in this study. Our analysis showed strong correlation between O2 saturation indices and OAHI (Table 5). Therefore, O2 indices do not correlate with REM‐OSA or REM‐AHI/NREM‐AHI ratio once OAHI is adjusted for.

We found that REM‐OSA group was younger than NREM‐OSA group (Table 1). However, the effect of age disappeared in the regression models after adjusting for BMI, because of the strong collinearity between age and BMI in the entire cohort (Table 5). Also, BMI per se was not an independent predictor of REM‐OSA or of high REM‐AHI/NREM‐AHI ratio. These findings further support that REM‐OSA and NREM‐OSA are not separate phenotypes. Previous studies showed higher frequency of REM‐OSA in younger patients. This is likely related to differences in study design, age distribution of included subjects and data analysis. Verhelst et al. and Zhang et al. have previously showed that REM‐OSA decline in prevalence with increasing age [17].

REM‐OSA was not affected by patients' underlying diagnosis which suggest that REM‐OSA is not related to specific clinical characteristics. We also found no gender differences between REM‐OSA and NREM‐OSA similar to previous pediatric studies that showed similar rates of REM and NREM‐OSA between males and females [1].

Our study is limited by its retrospective design and by its exclusion of mild OSA which is makes the generalization of its conclusion not justifiable in patients with mild OSA disease.

In conclusion, our study findings suggest that REM‐OSA and NREM‐OSA are not separate clinical phenotypes but only markers of OSA disease severity, with NREM‐OSA indicating more severe disease. These findings support the use of REM‐AHI/NREM‐AHI ratio as an additional parameter for OSA severity to conventional parameters (i.e. AHI, OAHI, REM‐OAHI) which can be helpful in clinical practice, PSG interpretation, treatment decisions and future studies. The effect of this ratio on treatment outcomes and long‐term clinical outcomes in pediatric OSA should be studied.

## Author Contributions

**Haneen Toma:** writing – original draft. **Amal Alnaimi:** methodology. **Mutasim Abu‐Hasan:** writing – review and editing. **Antonisamy Belavendra:** formal analysis. **Ibrahim Janahi:** supervision. All authors have approved current manuscript.

## Ethics Statement

The study was approved by the Research Ethics Board at Sidra Medicine, Doha, Qatar (IRB no 2202346). A waiver of informed consent was obtained because this was a retrospective observational study. All patient data were anonymous and personal identifiers were removed from the data collection forms.

## Conflicts of Interest

The authors declare no conflicts of interest.

## Data Availability

All data relevant to this article are available upon request.
